# Can portable tomosynthesis improve the diagnostic value of bedside chest X-ray in the intensive care unit? A proof of concept study

**DOI:** 10.1186/s41747-017-0021-6

**Published:** 2017-10-27

**Authors:** Jeroen Cant, Annemie Snoeckx, Gert Behiels, Paul M. Parizel, Jan Sijbers

**Affiliations:** 1 Imec - Vision Lab, Department of Physics, University of Antwerp, Groenenborgerlaan 171, 2020 Antwerp, Belgium; 2 Agfa HealthCare NV, Septestraat 27, 2640 Antwerp, Belgium; 3 Department of Radiology, University of Antwerp, Antwerp University Hospital, Wilrijkstraat 10, 2650 Antwerp, Belgium; 4 University of Antwerp, Groenenborgerlaan 171, 2020 Antwerp, Belgium

**Keywords:** Bedside chest X-ray, Digital tomosynthesis, Intensive care unit (ICU), Portable (mobile) technology, Thorax

## Abstract

Portable bedside chest X-ray (CXR) is an important and frequently used tool in the intensive care unit (ICU). Unfortunately, the diagnostic value of portable CXR is often low due to technical limitations and suboptimal patient positioning. Additionally, abnormalities in the chest may be hidden on the projection image by overlapping anatomy and devices such as endotracheal tubes, lines and catheters. Digital tomosynthesis (DTS) can solve the problem of anatomical overlap. In DTS, several low-dose X-ray images from different angles are acquired and subsequently used by a reconstruction algorithm to compute section images along planes parallel to the detector. However, a portable device to be used for portable bedside chest DTS is not on the market yet. In this work, we discuss modifications to a portable X-ray device to enable portable DTS and illustrate the potential of portable DTS to improve the diagnostic value of bedside CXR in the ICU. A simulation, based on computed tomography scans, is presented. Our experiments comparing portable DTS with conventional bedside CXR showed a substantially improved detection of pneumothorax and other abnormalities.

## Key points

- The diagnostic value of portable bedside chest X-ray (CXR) is often limited, especially in the ICU
- Digital tomosynthesis (DTS) offers separation of anatomical overlap in portable CXR into different section images
- A novel concept for a portable DTS device is described
- Portable DTS exams of ICU patients were simulated, based on computed tomography scans
- Simulated portable DTS showed potential to improve the diagnostic value of portable CXR

## Introduction

Chest X-ray (CXR) plays an important role in patient management in the intensive care unit (ICU) [1], assisting physicians in the diagnosis and follow-up of a variety of cardiopulmonary disorders. It is valuable for identification of findings necessitating emergency medical care, such as pneumothorax, (ventilator-associated) pneumonia or atelectasis, and assessment of volume status [2]. Additionally, CXR is used to evaluate a broad range of intrathoracic medical devices, ensuring proper positioning and surveying for complications [3–5]. Although the clinical usefulness for daily follow-up of patients with CXR in the ICU is under constant scrutiny [6, 7], the American College of Radiology considers the use of CXR in the ICU highly appropriate, especially upon admission and after placing invasive devices such as endotracheal tubes, endovascular catheters and drains, or when the condition of the patient worsens [8].

Bedside CXR is the imaging modality of choice for evaluation of the chest in ICU-patients, since these patients need to be monitored closely and are often less mobile due to mechanical ventilation or other medical devices such as drains and tubes. However, the diagnostic value of portable CXR in the ICU is significantly lower when compared to a CXR obtained with a dedicated wall-mounted flat panel device. First of all, compared to a standard posterior-anterior (PA) and lateral (LAT) exam, the portable anterior-posterior (AP) exam of a bedridden patient in supine position shows an apparently enlarged width of the heart, which may obscure retrocardiac structures, especially in poor inspiration. Also, the detection and assessment of certain abnormalities (e.g. pneumothorax) is known to be more difficult on portable CXR obtained in a reclining patient when compared to CXR acquired from an upright patient [9]. Additionally, the clinical condition of the patient often hinders optimal patient positioning, causing asymmetric images and superposition of anatomical structures (e.g. of the clavicles over the lung apices). Frequently, superposition of devices, tubes and catheters as well as patient related factors (obesity, hypoventilation, motion) further reduce the quality of the portable CXR in the ICU. Finally, the portable CXR is subject to technical limitations: images are acquired using a voltage of 90 kVp instead of 120 kVp, resulting in a less translucent appearance of the ribs. Image contrast in portable CXR is also reduced due to the absence of an anti-scatter grid while the reduced source-image distance of only 1.2 m causes higher geometrical distortions [10].

**Fig. 1:**
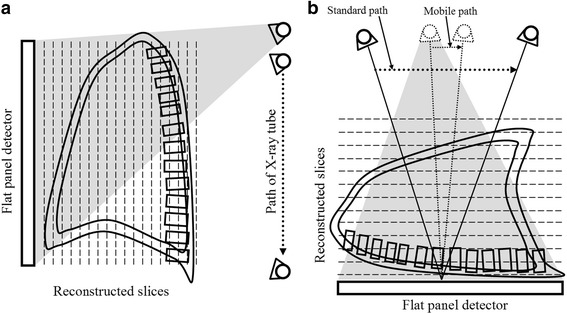
Chest digital tomosynthesis (DTS) with a wall mounted detector (**a**) and a portable bedside system (**b**). In both setups, a motorised X-ray tube moves on a (typically straight) path parallel to a stationary flat panel detector, producing several low dose posterior-anterior DTS projections. Next, coronal slices (*dashed lines*) are computed using a reconstruction algorithm. Note the reduced X-ray tube path length for the acquisition of portable anterior-posterior DTS projections of a bedridden supine patient in **b**

Digital tomosynthesis (DTS) has potential to improve the diagnostic value of bedside CXR, in particular the depth resolution. Chest DTS is a technique already available on a wall-mounted flat panel or X-ray table [11]. First, a small number of low-dose X-ray images (usually between 35 and 60) are acquired with a motorised X-ray source, which moves relative to a stationary detector, as illustrated in Fig. 1a. Next, advanced reconstruction algorithms are used to compute coronal section images. These section images have a higher in-plane resolution than computed tomography (CT) scans, but a lower depth resolution of each slice, due to the limited sweep angle of the X-ray tube (typically 30°) [12]. Thanks to its ability to separate overlapping anatomical structures into subsequent slices, chest DTS has been reported to improve the detection of lung nodules compared to conventional CXR [13–15] and on visualising other findings related to various pulmonary diseases [16, 17]. Additionally, chest DTS has the potential to optimise the use of CT resources and reduce the effective radiation dose to the patient population [18, 19]. Therefore, portable chest DTS could also substantially increase the diagnostic value of portable CXR in the ICU, even though currently no portable version of a chest DTS modality is available.

## Portable tomosynthesis simulation

A portable device for DTS does not exist yet. In our simulation, we assume a portable X-ray modality is used to acquire several X-ray projections from different angles. The X-ray tube moves in a motorised way along a straight path. Alternatively, a system could be designed without motion, using a linear array of carbon nanotube sources. In our simulation, the source-image distance was set to 120 cm and the total tube travel distance was set to 14 cm. This corresponds to an angular range of 6° (substantially less than the typical 35° angular range in chest DTS with a dedicated wall-mounted flat panel detector) to reduce the risk of collision with bedside equipment and simplify the design of the device.

We retrospectively selected two ICU patients from the hospital Picture Archiving and Communication System (University Hospital of Antwerp) who showed abnormal findings on CXR and underwent a chest CT scan within a time frame of 1 h. CT scans had been obtained using a 64-slice unit (LightSpeed VCT, GE Medical Systems, US) with the following technical parameters: 99 mA, 120kVp, 600 ms. The need for informed consent was waived by the Ethical Committee. From the anonymised CT data, which consisted of voxels of 0.5 × 0.5 × 0.84 mm size, 15 DTS projections were simulated using the ASTRA tomography toolbox (v1.6, Antwerp, Belgium) [20], which contains algorithms for simulating X-ray radiographs from volumetric data. Simulated DTS projections were computed by a linear forward projection of the CT volume onto a virtual flat panel detector, and contained 831 × 1012 pixels of 0.42 mm size. No scatter or electronic noise were simulated. Next, from the simulated DTS projections, 55 coronal section images of 5-mm depth were reconstructed using 50 iterations with the simultaneous iterative reconstruction technique (SIRT) [21]. Finally, the reconstructed DTS section images were compared to the original CXR and interpreted for ICU-related abnormalities. The effective dose of the portable chest DTS exam, as described in this study, was computed using the PCXMC [22] simulation software.

To validate this DTS simulation based on CT scans, a simulated DTS exam was compared with an experimentally acquired DTS exam of an anthropomorphic chest phantom (Humanoid Systems, Carson, USA). For the experimental DTS exam, 15 chest DTS projections were acquired manually (i.e. without motorised tube motion) with a mobile X-ray unit (Agfa Healthcare DXD-100, Mortsel, Belgium), from the same angles and source image distance as in the ICU simulations above. All DTS projections were obtained at 90 kVp and 0.1 mAs, which was the lowest possible tube current of the mobile unit. Subsequently, 55 coronal section images of 5-mm depth were computed by DTS using 30 SIRT iterations. For the simulated DTS exam, 15 DTS projections were simulated from a CT scan of the anthropomorphic phantom and a simulated DTS reconstruction was computed. Reconstructions from the experimental DTS exam and CT based simulated DTS exam were compared visually by a radiologist with 10 years’ experience in thoracic imaging.

## Results

**Fig. 2:**
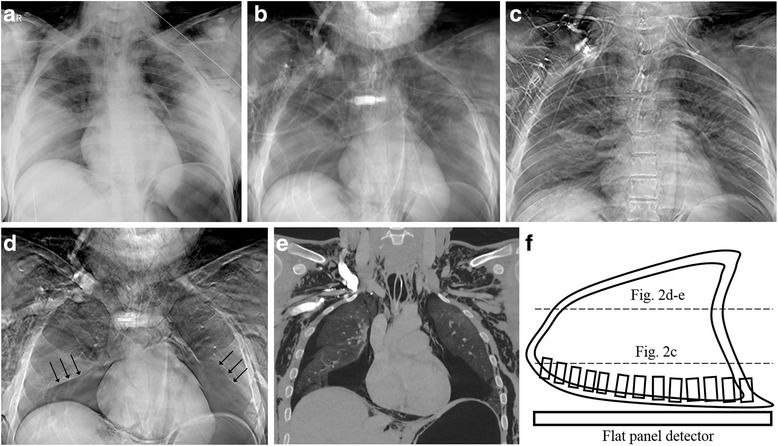
Bedside CXR and simulated DTS reconstruction for a 26-year old woman, who was admitted to the ICU after a cardiac arrest. Shortly after admission, she developed severe subcutaneous emphysema. **a** The bedside CXR confirms subcutaneous emphysema in the neck, axillae and lateral chest walls. The symmetric dense areas which overly the midsection of both lung fields are caused by bilateral breast prosthesis. There is no deep sulcus sign, and no clear delineation of a pneumothorax. The left diaphragm is clearly delineated due to a large amount of air in the stomach. The heart contour is relatively sharp, but symmetric on both sides. **b** Simulated DTS acquisition of a CT examination, performed 30 minutes later. **c** Slice of the DTS reconstruction of the simulated acquisitions near the back of the patient shows symmetric findings with normal parenchyma on both sides, without areas of consolidation or hyperlucency. **d** Simulated DTS slice more anterior than that shown in **c** shows two triangular areas with absence of lung markings; these findings are consistent with a bilateral pneumothorax. Note that these areas have a similar translucency compared to the large amount of air in the stomach. **e** Coronal reformatted CT image at the same level of the image shown in **d**, confirming a bilateral pneumothorax and extensive subcutaneous emphysema. **f** Slice locations of the DTS and CT slices

The results of the simulated portable DTS exam are displayed in Fig. 2 for the first selected ICU patient. Whereas the bedside CXR shows no clear delineation of a pneumothorax (Fig. 2a), the more anterior simulated DTS slice (Fig. 2d) shows triangular areas with absence of lung parenchyma, which are consistent with bilateral pneumothorax.

**Fig. 3:**
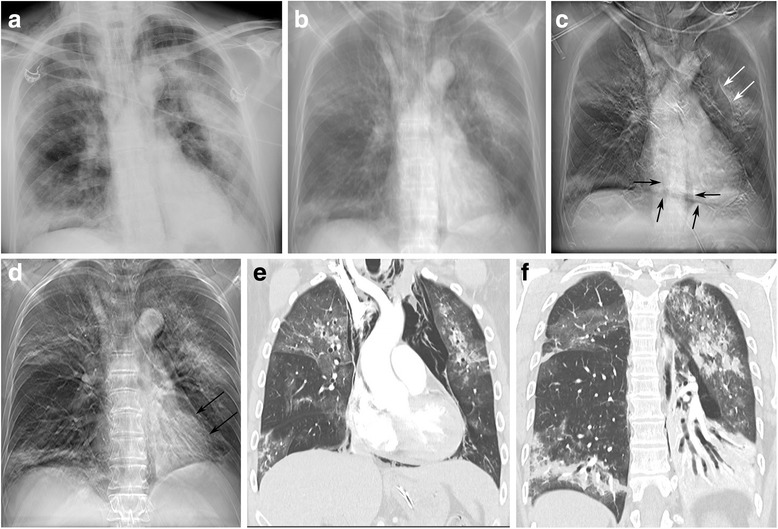
Bedside CXR and simulated DTS reconstruction of a 49-year-old man who was admitted to the ICU after a neurosurgical intervention with removal of a fourth ventricle ependymoma. Five days after surgery he developed respiratory insufficiency. **a** Bedside CXR shows subcutaneous emphysema in the neck and pulmonary consolidations bilaterally in the basal parts of both lungs. **b** Simulated DTS acquisition from a CT examination, performed 1 h later. Because of the discrepancy between respiratory function and findings on CXR, the CT examination was performed to rule out pulmonary embolism. **c** Slice of the DTS reconstruction of the simulated acquisitions at the level of the heart shows linear areas of hyperlucency around the heart and in the paravertebral region (*black arrows*). Also note an area of relative hyperlucency in the left lung with a clear delineation of the adjacent consolidation (*white arrows*). These findings are consistent with pneumomediastinum. **d** Slice of the DTS reconstruction of the simulated acquisitions more posterior than DTS reconstruction A shown in **c**, showing a large retrocardiac consolidation with air bronchogram (*black arrows*). **e** A coronal reformatted CT image confirms the pneumomediastinum and a large consolidation (**f**) with air bronchogram, consistent with subtotal atelectasis of the left lower lobe. Due to the retrocardiac location, the consolidation is not visible on the CXR. Moreover, the extent of the pneumomediastinum is better appreciated on the DTS reconstructions compared to the bedside radiograph

For the second selected ICU patient, a CT was performed to rule out pulmonary embolism because of the discrepancy between respiratory function and findings on CXR. Due to the retrocardiac location, a consolidation detected on the DTS slices (Fig. 3c, d) was not visible on the CXR (Fig. 3a). Moreover, the extent of the pneumomediastinum could be better appreciated on the DTS reconstructions.

**Fig. 4:**
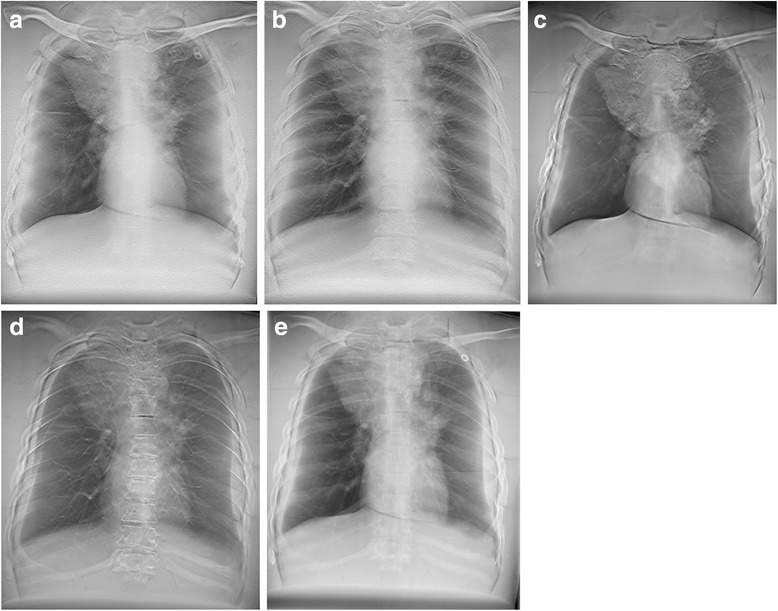
**a, b** Experimentally acquired portable DTS slices of a chest phantom, located at two different coronal planes. **c, d** Simulated DTS slices from a multi-slice CT examination of the same phantom. Note the similar depth resolution between experimental and simulated DTS. Although the simulated DTS slices show a higher visual contrast, all structures present in the simulated images can also be appreciated in the experimentally acquired DTS slices. **e** A standard radiograph of the chest phantom for comparison

The resulting DTS reconstruction from the experimentally acquired images of the anthropomorphic phantom are displayed in Fig. 4, together with the simulated DTS reconstructions from a CT scan. Image contrast in the experimentally acquired DTS reconstructions appears lower than that in the simulated counterpart, as the simulations did not include scatter. However, despite differences in visual appearance and small discrepancies between slice location, both acquired and simulated DTS showed similar structures and level of depth resolution. The dose simulation of the portable DTS exam resulted in an effective dose of 0.029 mSv.

## Discussion

In this work we describe a new modality for bedside chest DTS. In the presented simulations of ICU patients, portable DTS improved the detection of abnormalities in the bedside CXR. Whereas the clinical use of chest DTS for patients in the radiology department can be evaluated against CT, MRI and other modalities, far fewer alternatives exist for bedside chest examination of patients in the ICU, for whom transport to a radiology department is often complicated and expensive. The cost of performing bedside ultrasound as a daily follow-up is relatively high, since this exam takes longer to perform and requires a high level of expertise. Mobile CT scanners exist but are not widespread and they are mainly limited to dedicated applications such as imaging of the head [23–25]. Portable chest DTS could be implemented on a mobile X-ray device, which is already widely accepted as a mobile examination device. Portable DTS might also hold potential to improve bedside exams in the emergency room, sterile rooms in haematology wards or transplantation units, burn units, surgical departments, etc. In future work, a more elaborate prospective clinical study of potential applications will be conducted, in combination with an optimisation of the acquisition technique.

The radiation dose for a chest DTS exam using a dedicated wall-mounted flat panel is around 0.12 mSv for a typical acquisition of 60 projections [26]. In our experiment, however, only 15 DTS projections were taken at 90 kVp and 0.1 mAs. The effective dose of 0.029 mSv corresponds to approximately 1.5 times the dose of a portable CXR, obtained at 90 kV and 1 mAs.

Söderman et al. [27] showed that decreasing the tube travel distance, and thus the angular range, has a positive effect on the reproduction of the trachea and paratracheal tissue, vessels, and aorta. However, a smaller angular range resulted in decreased image quality related to following vessels through the volume. Image quality for findings more specifically related to critically ill patients, such as pneumothorax/pneumomediastinum, and the capability of separating overlapping devices into different slices were not discussed. Also, the aforementioned study investigated different configuration parameters for chest DTS with an effective dose comparable to a standard DTS exam. Our simulations showed portable chest DTS in the ICU with a small angular scanning range (only 6°) and a radiation dose of only 0.029 mSv. The effective slice thickness of the simulations presented in this study was obtained experimentally by adding a thin line throughout the simulated patient from head-left-posterior to foot-right-anterior. The amount of this line that was visible in the reconstructed tomosynthesis slices illustrated an effective slice thickness corresponding to a patient slab of 30 mm. Despite this relatively high effective slice thickness, the diagnostic quality of the bedside CXR was improved.

The presented simulation study has obvious limitations. First, the quality of the bedside DTS reconstruction relies heavily on the accuracy of the measured relative positions and orientation of the X-ray source trajectory and detector, which was assumed to be error-free in our simulation. In a real-life acquisition, our previous work could be applied to correct possible misalignment [28, 29]. Secondly, patient motion during the acquisition of the DTS projections is another well-known cause of degradation of DTS image quality, which was not included in our simulation. Despite a reduced total acquisition time of a few seconds, due to the reduced number of 15 exposures, breathing or other types of patient motion might still occur. Many ICU patients might not be able to hold their breath, although for intubated patients, a short interruption in mechanical ventilation could be considered in some cases. Reconstruction and motion correction methods might need to be incorporated and improved in a portable chest DTS device [30, 31]. Finally, physical phenomena such as scatter and quantum noise were excluded from these preliminary simulations. However, the reconstructions from the experimentally acquired DTS exam of the humanoid phantom strengthen our belief that the conducted experiments are at least indicative of the expected performance of mobile chest DTS.

In conclusion, we have shown with preliminary simulations that portable chest DTS holds potential to improve the diagnostic accuracy of bedside CXR in the ICU. Possible benefits include: improved localisation of parenchymal consolidations (anterior versus posterior), detection of pneumothorax or pneumomediastinum patients in the supine position, verification of the correct position of drains and lines, differentiation between pleural effusions and consolidations, and other applications. It is technically feasible to perform mobile chest DTS with a modified mobile X-ray unit, which is already widely accepted as a mobile examination tool.
